# GSATools: analysis of allosteric communication and functional local motions using a structural alphabet

**DOI:** 10.1093/bioinformatics/btt326

**Published:** 2013-06-05

**Authors:** Alessandro Pandini, Arianna Fornili, Franca Fraternali, Jens Kleinjung

**Affiliations:** ^1^Randall Division of Cell and Molecular Biophysics, King’s College London, London SE1 1UL, ^2^The Thomas Young Centre for Theory and Simulation of Materials, London WC2R 2LS and ^3^Division of Mathematical Biology, MRC National Institute for Medical Research, London NW7 1AA, UK

## Abstract

**Motivation:** GSATools is a free software package to analyze conformational ensembles and to detect functional motions in proteins by means of a structural alphabet. The software integrates with the widely used GROMACS simulation package and can generate a range of graphical outputs. Three applications can be supported: (i) investigation of the conformational variability of local structures; (ii) detection of allosteric communication; and (iii) identification of local regions that are critical for global functional motions. These analyses provide insights into the dynamics of proteins and allow for targeted design of functional mutants in theoretical and experimental studies.

**Availability:** The C source code of the GSATools, along with a set of pre-compiled binaries, is freely available under GNU General Public License from http://mathbio.nimr.mrc.ac.uk/wiki/GSATools.

**Contact:** alessandro.pandini@kcl.ac.uk or jkleinj@nimr.mrc.ac.uk

**Supplementary information:** Supplementary data are available at *Bioinformatics* online.

## 1 INTRODUCTION

Biomolecular motions play a key role in several biological functions: enzymatic activity, protein–protein interactions, ligand binding and allosteric regulation. Computational approaches, such as molecular dynamics (MD), are now routinely used to reproduce the intrinsic dynamics of proteins, but effective tools are still required to gain functional insight from the simulated data.

Global collective motions are often associated with biological functions, and it was demonstrated that these motions can be extracted from conformational ensembles (Amadei *et al.*, 1993).

In a previous study, we suggested a method aimed at recovering the role of local conformational changes in functional motions (Pandini *et al.*, 2010). To this purpose, we developed a structural alphabet (SA): a set of 25 canonical states of four-residue protein fragments (C^α^ atoms only) describing the most probable local conformations in high-resolution protein structures. Therefore, the SA provides a means for the coarse-grained annotation and processing of local conformations in a string format, which lends itself to a range of efficient sequence analysis algorithms.

The SA has been used successfully in analyzing local changes in implicit solvent simulations (Kleinjung *et al.*, 2012), allosteric signal transmission (Pandini *et al.*, 2012) and conformational changes on effector binding (Baussand and Kleinjung, 2012).

Here, we present GSATools, a set of SA-related tools interfacing with GROMACS (Pronk *et al.*, 2013) for the analysis of conformational ensembles. GSATools is a software package designed for the investigation of the conformational dynamics of local structures, the functional correlations between local and global motions and the mechanisms of allosteric communication.

## 2 IMPLEMENTATION AND FUNCTIONALITY

GSATools was implemented in C as a set of analysis programs for GROMACS 4.0.x (Van Der Spoel *et al.*, 2005) and 4.5.x (Pronk *et al.*, 2013) with a user-friendly and familiar interface. The required input is a trajectory file with a reference structure of the protein in PDB format. The trajectory can be composed of conformers derived by MD or other simulation methods. The analysis is performed at the C^α^ level so that ensembles from one-bead coarse-grained methods are also suitable input.

The requirement to install GSATools is a working installation of GROMACS. GSATools comprises the *g_sa_encode* program to encode a protein trajectory into an alignment of structural strings and to perform basic statistical analysis, and the *g_sa_analyze* program to perform correlation analysis. The software distribution includes exemplary R (R-Development Core Team, 2010) scripts to directly generate figures and plots from the output files. In addition to standard R libraries, some scripts require the Bio3D package (Grant *et al.*, 2006).

### 2.1 SA encoding and analysis of local motions

The dynamics of local structures is captured by comparison of the sampled conformations with a set of representative backbone fragments (Pandini *et al.*, 2010). The conformation of a protein of *n* residues is condensed in a structural string of length *n-3* (Pandini *et al.*, 2007); therefore, an MD ensemble containing *m* conformers can be encoded into a set of *m* aligned structural strings. This structural alphabet (SA) alignment can be saved in FASTA format for further sequence-oriented analyses.

**Fig. 1.**
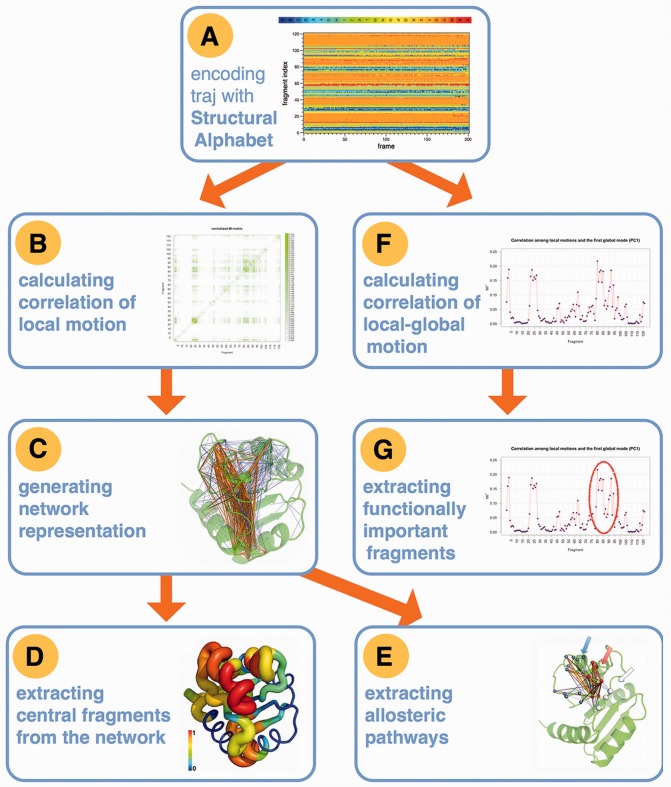
Overview of the major analysis steps for the NtrC protein (Pandini *et al.*, 2012). A high-resolution image is given in the Supplementary Data

The *g_sa_encode* program provides two modes of encoding [local and global (Park and Levitt, 1995)] and yields several statistics metrics about the encoded trajectory. The user can visualize the time evolution of structural changes by a color-coded representation of the alignment (Fig. 1A). Additionally, the accuracy of the encoding can be measured and plotted for inspection. The extent of conformational variability at each position can be estimated by the Shannon entropy, and the relative frequency of the representative fragments at each position can be saved as a sequence profile of the alignment. Example graphs of these statistics are included in the Supplementary Data.

The condensed string representation and the sequence statistics are particularly suitable to detect subtle conformational changes often hidden by analyses of global structure and dynamics (Pandini *et al.*, 2012).

### 2.2 Analysis of correlated local motions and allostery

The correlation between local conformational changes of two fragments in a protein can be calculated as the mutual information (MI) between two columns (positions) in the SA alignment. The MI matrix (Fig. 1B) of all pairwise correlations is a concise representation of a protein’s local motions (Pandini *et al.*, 2012). The correlation analysis is performed using the *g_sa_analyze* on the SA alignment. The program calculates the positional MI matrix and estimates the statistical significance of each correlation. Additionally, a transition probability matrix can be calculated to estimate the relative frequency of specific fragment transitions.

A network model of the local motions can be derived from the normalized MI matrix. The software distribution includes an R script to output a GML file for visualization in Cytoscape (Shannon *et al.*, 2003) and a PyMOL (Schrödinger, 2009) plug-in to project the network onto the protein structure (Fig. 1C).

Key protein fragments can be identified by eigenvector centrality (Newman, 2010) within the correlation network using a provided R script. Nodes with higher network centrality represent fragments that show correlated motions preferentially with other highly correlated fragments (Fig. 1D).

If the protein of interest has an allosteric function, communication pathways between the allosteric and orthosteric sites can be extracted from the network model as shown in Figure 1E (Pandini *et al.*, 2012).

### 2.3 Detection of local motions correlated to function

Functionally relevant motions can be identified if a function-related structural property is known. Generally, global or collective motions are considered for this type of analysis (Hub and De Groot, 2009). In the GSATools approach, the *g_sa_analyze* scans the protein to detect contributions to a functional change by local motions.

Any time-dependent index of a function-related property can be provided. Then the *g_sa_analyze* program can calculate the correlation (as MI) between the functional index and each protein fragment (Fig. 1F). The MI value is eventually used as a score to predict putative regions of the protein for further analysis, e.g. site-directed mutagenesis (Fig. 1G).

The functional index may be represented by a collective motion known to be associated with a biological mechanism (Pandini *et al.*, 2012). In this special case, the analysis can identify local structures (e.g. hinges) whose motion is propagated to trigger a functional change on a global scale.

### 2.4 Tutorial

The software distribution includes a step-by-step tutorial (Supplementary Data) with input files, output files, shell scripts and R scripts. The scripts can easily be modified to process user-provided input data.

## 3 CONCLUSIONS

GSATools is a free, easy-to-use and fully documented software for the analysis of conformational ensembles of proteins. The GSATools complements the GROMACS toolkit with a powerful set of analyses to detect, annotate and interpret local motions of functional relevance.

Dynamics of local structures, functional correlations of local and global motions and mechanisms of allosteric communication can be extracted from ensembles of conformations. An example of a comprehensive analysis is provided in the software distribution; each analysis can be performed independently if desired. Results are generated in standard formats for easy comparison with other analyses aimed at identification of global motions, i.e. principal component analysis (Amadei *et al.*, 1993).

GSATools addresses the need for automated functional analysis emerging from the wealth of molecular simulations currently available in the scientific community.

*Funding*: This research was supported by the Medical Research Council (U117581331 to JK); the British Heart Foundation (FS/12/41/29724 to AF); the Biotechnology and Biological Sciences Research Council (BB/I023291/1 and BB/H018409/1 to AP and FF).

*Conflict of Interest*: none declared.

## Supplementary Material

Supplementary Data
