# Supplementary material for: GSATools: analysis of allosteric communication and functional local motions using a structural alphabet
Source: Bioinformatics. 2013 Jun 5;29(16):2053–5. doi: 10.1093/bioinformatics/btt326 (PMC3722520; doi:10.1093/bioinformatics/btt326)
Supplement: Supplementary Data [file supp_29_16_2053__index.html]

GSATools: analysis of allosteric communication and functional local motions using a Structural Alphabet — GSATools: analysis of allosteric communication and functional local motions using a structural alphabet — GSATools: analysis of allosteric communication and functional local motions using a structural alphabet — Supplementary Data 

# GSATools: analysis of allosteric communication and functional local motions using a structural alphabet

## 

files

**Files in this Data Supplement:**

- Supplementary Data - pdf file
- Supplementary Data - tiff file
