# Supplementary material for: GSATools: analysis of allosteric communication and functional local motions using a structural alphabet
Source: Bioinformatics. 2013 Jun 5;29(16):2053–5. doi: 10.1093/bioinformatics/btt326 (PMC3722520; doi:10.1093/bioinformatics/btt326)
Supplement: Supplementary Data [file supp_btt326_APandini_SupplementaryData_Tutorial.pdf]

Supplementary Data

GSATools: analysis of allosteric communication and functional local motions using a Structural Alphabet

Alessandro Pandini*, Arianna Fornili, Franca Fraternali and Jens Kleinjung*

This step-by-step tutorial describes the installation and main functionalities of GSATools. A directory tree with all the necessary files and shell scripts is available in the software distribution:

```
gsatools-4.0.x-1.00
└──tutorial
         ├── 00_input_data
         ├── 01_SA_encoding
         ├── 02_SA_statistics
         ├── 03_local_correlation
         ├── 04_network_analysis
         └── 05_functional_analysis
```

Additionally, the main directory and each subdirectory contain a script to automatically run all the associated analyses (i.e. run_tutorial.sh to run all the exercises, 01_SA_encoding.sh to run only the first, etc). The user can either type the commands for each exercise or run the corresponding script. All the scripts can be easily modified to process user-provided data by changing the parameters in the first sections of the files.
Typographic convention: commands are prepended by the > sign.

The protein studied in this tutorial is the receiver domain of the response regulator NtrC, Nitrogen regulatory protein C (Pandini, et al., 2012). Please refer to the main text and references therein for an explanation of the theory behind the calculations here presented and for a discussion of allosteric modulation in NtrC.

GSATools has been designed to highlight correlations in the dynamics of backbone fragments, so that side chain motions and the overall protein collective motions are only implicitly taken into account through their effect on fragment motions. Methods that explicitly consider side chains (Ghosh and Vishveshwara, 2007) or global position vectors of atoms (Ghosh and Vishveshwara, 2007; Fanelli and Seeber, 2010; Morra, et al., 2009; Genoni et al., 2010; Chennubhotla and Bahar, 2007; Sethi, et al., 2009) in the calculation of correlations can be used to complement the present approach and to assess the relative importance of these types of motions in the system under study.

Installation

The following instructions assume GROMACS installed in the /usr/local directory and a terminal with bash shell. The GSATools are provided for GROMACS 4.0.x (Van Der Spoel, et al, 2005) and 4.5.x (Pronk, et al., 2013), please make sure to use the appropriate version of the software (here for convenience 4.0.x is assumed but the tutorial is included in both versions) and that GROMACS was not compiled for MPI.

The GSATools requires the following software:

- gromacs
- gsl-devel

Additionally to perform this tutorial it is required the installation of the following software:

- R http://www.r-project.org (R-Development-Core-Team, 2010)
- igraph http://cran.r-project.org
- qvalue http://www.bioconductor.org/packages/release/bioc/html/qvalue.html
- bio3d http://thegrantlab.org/bio3d (Grant, et al., 2006)

To compile the GSATools:

```
> tar xvfz gsatools-4.0.x-1.00.tar.gz
> cd gsatools-4.0.x-1.00
> export GMXDIR=/usr/local/gromacs
```

(If GROMACS is installed in a different location, please update the GMXDIR variable accordingly)

```
> ./configure
> make
```

After successful compilation the installation should be tested with the included checks:

```
> make check
```

If all 28 tests are successfully passed, the GSATools are ready to be used. The scripts/GSATOOLSRC.bash (or the equivalent for your shell) should be sourced to set the correct GROMACS environment variables:

```
> source scripts/GSATOOLSRC.bash
```

01 SA encoding

This exercise shows how to encode an MD trajectory into an alignment of structural strings and to generate the output in plain text, FASTA and X PixMap format. The XPM file is then converted in encapsulated postscript (eps) and Portable Document Format (pdf). Additionally, a measure of the encoding quality is recorded as RMSD of each letter in the encoding string from the corresponding fragment in the structure. A fragment is defined by 4 consecutive C^{α} atoms. A letter refers to the corresponding representative fragment in the Structural Alphabet (Pandini, et al., 2010).

```
> cd 01_SA_encoding
> ../../src/g_sa_encode -f ../00_input_data/T00.xtc -s ../00_input_data/T00.tpr -strlf
  T01.lf_str.out -rmsdlf T01.lf_rmsd.xvg -xpmlf T01.lf.xpm -fasta -xpm -log T01.log
```

This generates a few output files, namely:

- T01.lf_str.out alignment of structural strings (plain text)
- T01.lf_str.fasta alignment of structural strings (FASTA format)
- T01.lf.xpm image of the alignment (XPM format)
- T01.lf_rmsd.xvg RMSD distribution per position (XVG xmgrace format)

The encoding can be performed on a part of the trajectory (see command line flags `-b -e`) or only on frames at regular interval in time (see command line flag `-dt`). In this example the full trajectory is encoded.

The .xpm file can be conveniently converted into a postscript format using the GROMACS xpm2ps tool:

```
> xpm2ps -f T01.lf.xpm -o T01.lf.eps -legend none -di 01_ps.m2p
```

Then the .eps file can be converted into a .pdf with ps2pdf or read with an image editing program.

```
> ps2pdf T01.lf.eps
```

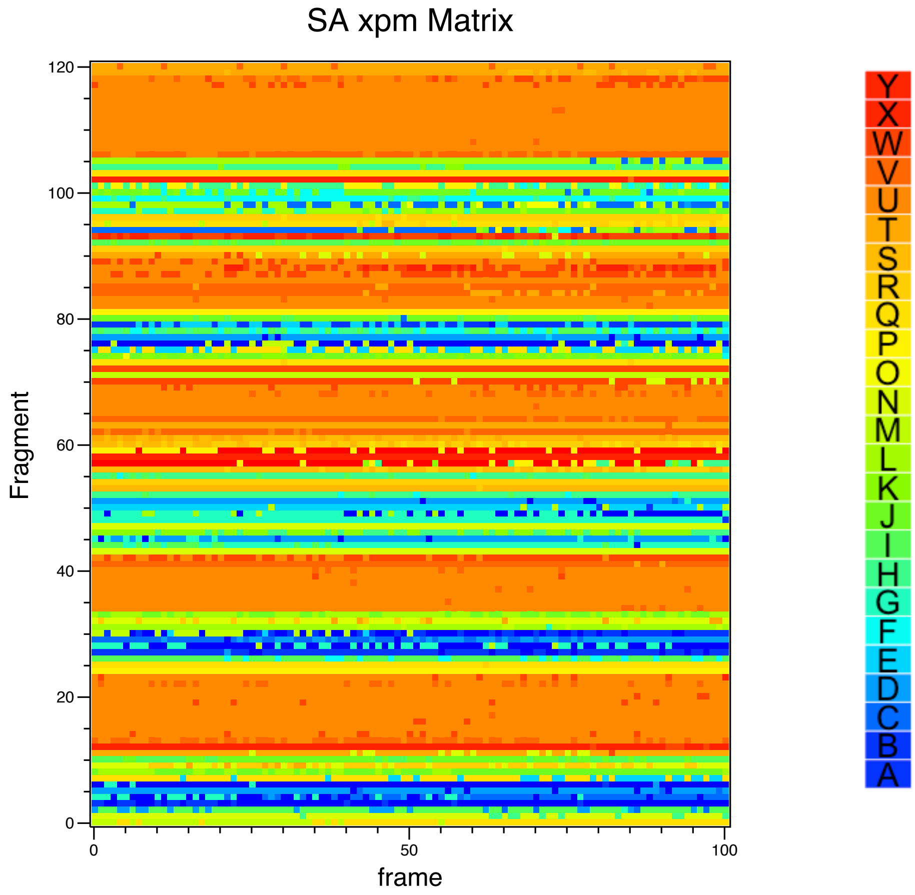

T01.lf.pdf (generated with ps2pdf)

A plot can be generated for the RMSD distribution per position.

```
> R CMD BATCH 01_plot_RMSD.R
```

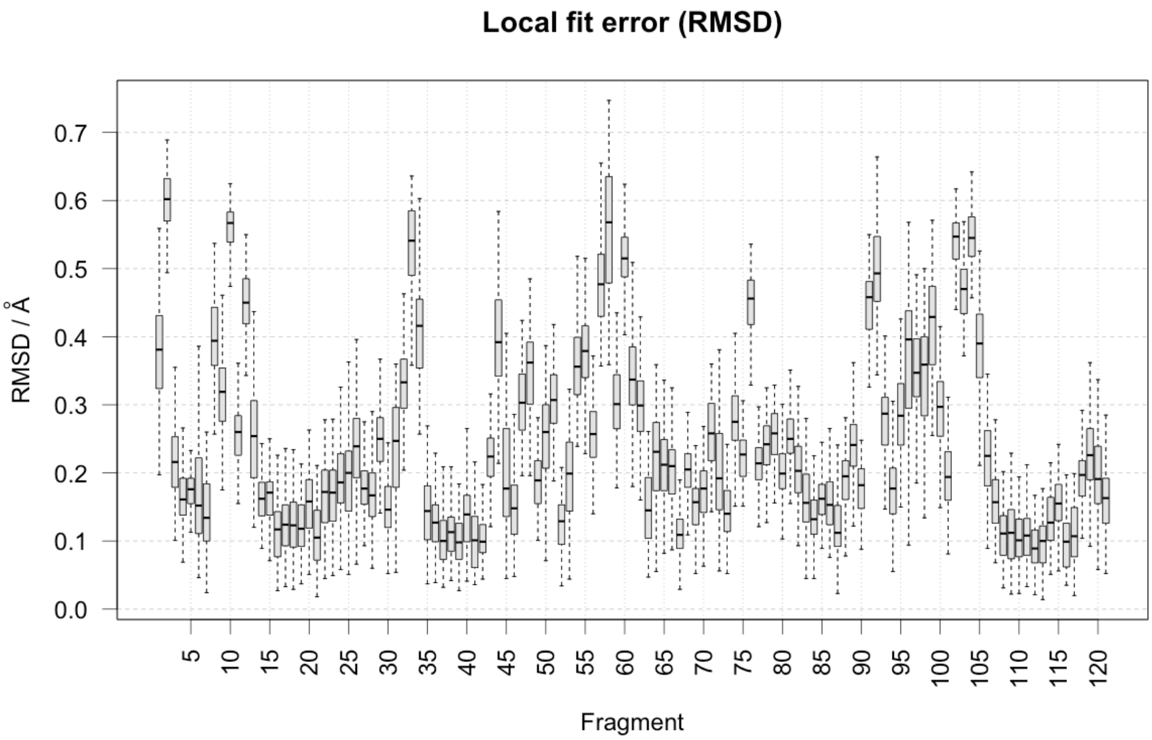

T01.lf_rmsd.png (generated with 01_plot_RMSD.R)

02 SA statistics

This exercise shows how to perform basic statistical analyses on the alignment of structural strings. It is possible to calculate the Shannon entropy per position, the sequence profile of the alignment and the transition probability matrix for the transitions between SA letters, which reflect local conformational changes.

```
> cd 02_SA_statistics
> ../../src/g_sa_analyze -sa T00.lf_str.out -H T02.lf_entropy.xvg -pro T02.lf_prof.dat -trans
  T02.lf_transmat.out -profile -entropy -trmat
```

This generates a few output files, namely:
- T02.lf_entropy.xvg the Shannon entropy per position (XVG xmgrace format)
- T02.lf_prof.dat sequence profile table (plain text)
- T02.lf_transmat.out transition probability matrix (plain text)

Plots can be generated for each output.

```
> R CMD BATCH 02_plot_entropy.R
> R CMD BATCH 02_plot_profile.R
> R CMD BATCH 02_plot_transmat.R
```

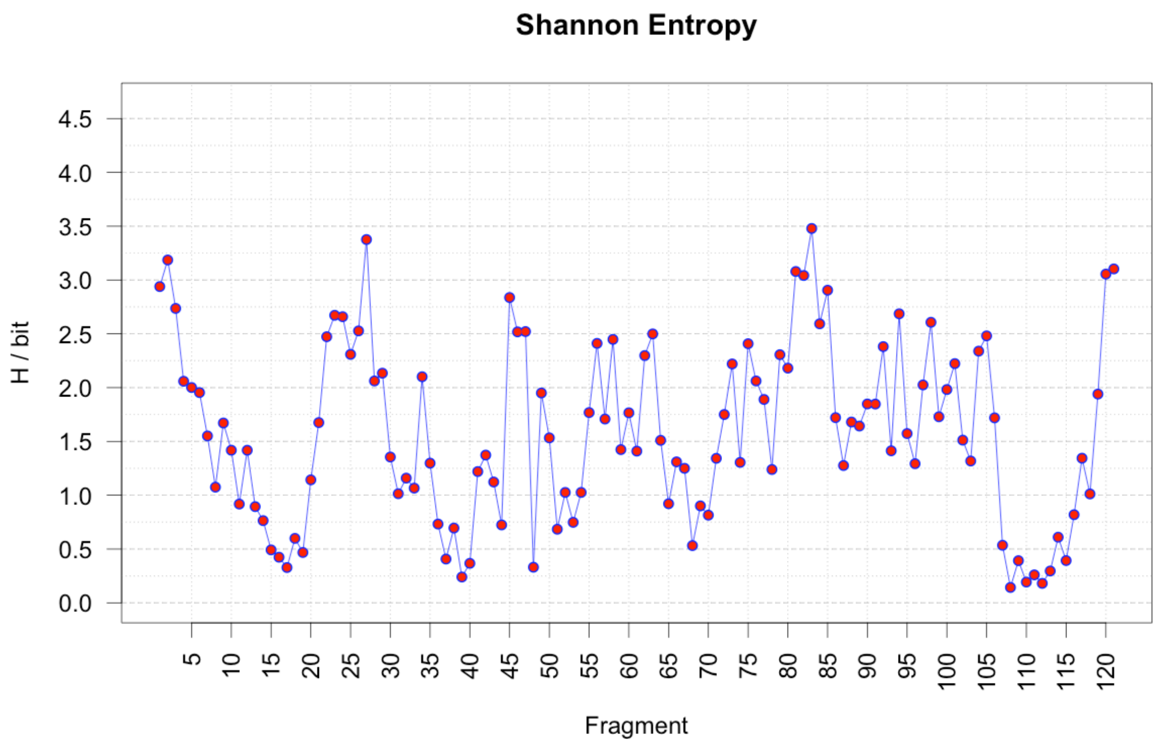

T02.lf_entropy.png (generated with 02_plot_entropy.R)

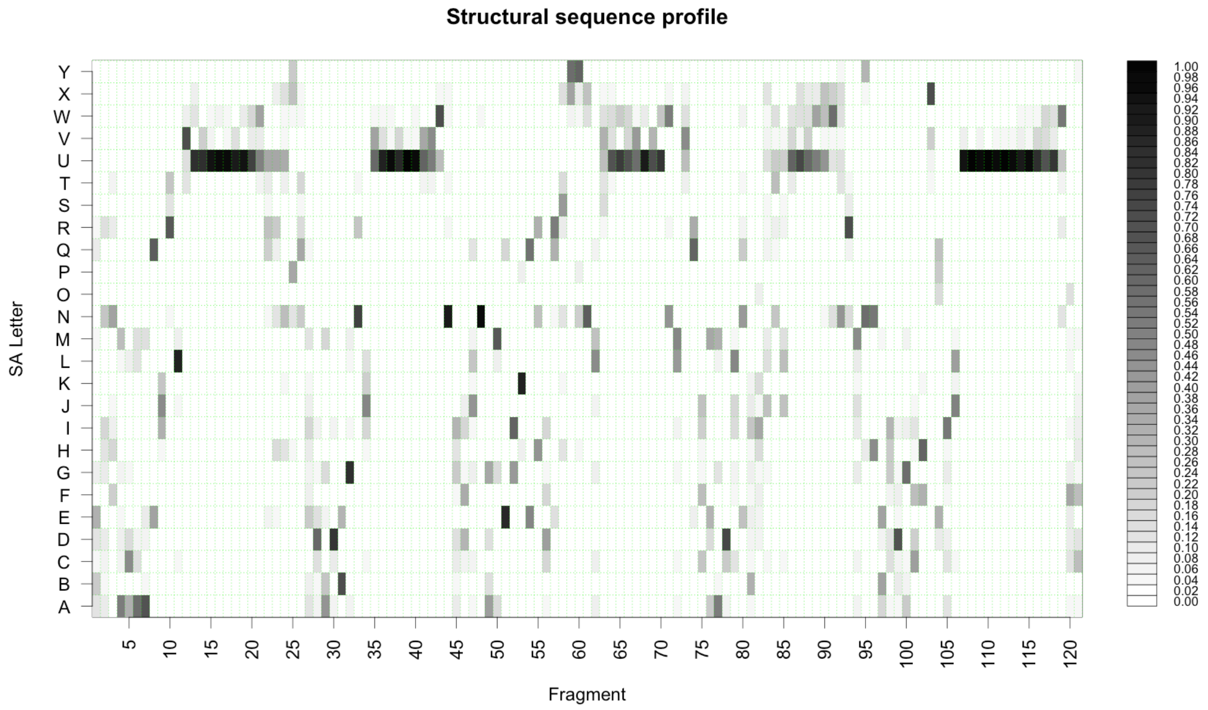

T02.lf_prof.png (generated with 02_plot_profile.R)

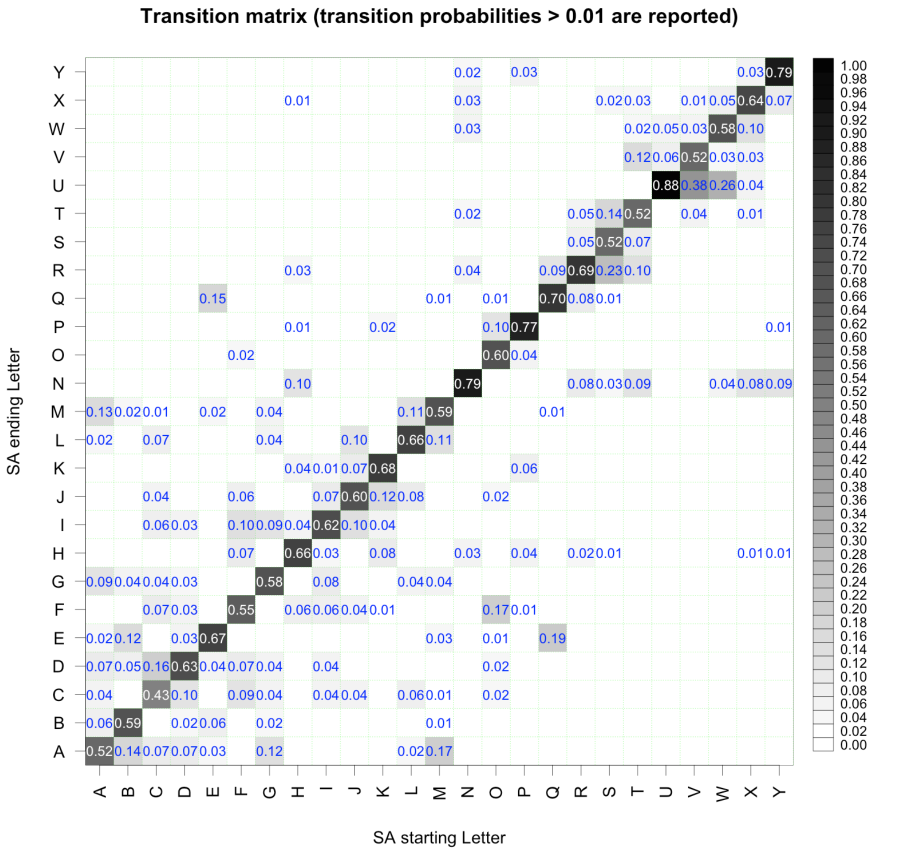

T02.lf_transmat.png (generated with 02_plot_transmat.R)

The columns of the matrix in the figure sum up to 1 so that the total probability of transition from a given starting letter to any letter is equal to 1.

03 local correlation

This exercise shows how to generate the Mutual Information (MI) matrix describing the correlation of local conformational changes among the protein's fragments. It is also possible to calculate the expected average error due to finite size sampling, the Joint Entropy and the normalized MI matrix. Details on the definitions and on the statistical analysis are available in (Pandini, et al., 2012).

```
> cd 03_local_correlation
> ../../src/g_sa_analyze -sa ../02_SA_statistics/T00.lf_str.out -MImat T03.lf_MImat.out -eeMImat
  T03.lf_eeMImat.out -jHmat T03.lf_jHmat.out -nMImat T03.lf_nMImat.out -MImatrix
```

This generates a few output files, namely:

- T03.lf_MImat.out MI matrix (plain text)
- T03.lf_jHmat.out Joint Entropy (H) matrix (plain text)
- T03.lf_nMImat.out normalised matrix calculated as MI / H (plain text)
- T03.lf_eeMImat.out finite-size error matrix (plain text)

A plot can be generated for the normalised MI matrix.

```
> R CMD BATCH 03_plot_nMI_matrix.R
```

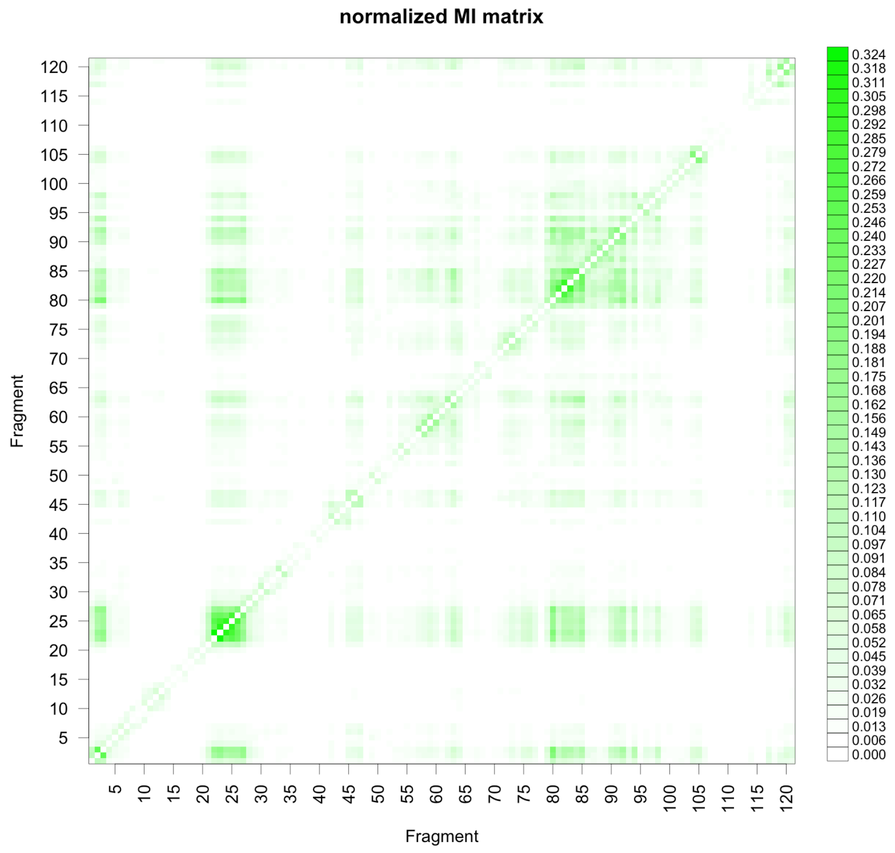

T03.lf_nMImat.png (generated with 03_plot_nMI_matrix.R)

04 networks analysis

This exercise shows how to generate a graph representation of the correlated local motions from the MI matrix (Pandini, et al., 2012). The R script requires also the Joint Entropy matrix, the expected average error due to finite size sampling and the statistical significance of each correlation. These input data are generated using *g_sa_analyze* (see exercise 03 local correlation).
The R script generates also an output for visualization in Cytoscape (Shannon *et al.*, 2003).

```
> cd 04_network_analysis
> R CMD BATCH 04_network_analysis.R
```

This generates a few output files, namely:

- T04.LF_enpdMI.mat	filtered MI matrix for network generation (plain text)
- T04.LF.cys.gml	Cytoscape readable network file (GML Graph Modeling Language format)
- T04.LF.evcent.dat	eigenvector centrality data file (plain text)

From the graph representation it is possible to calculate all the indices commonly used in network analysis. An example is the eigenvector centrality, which describes the relative importance of the nodes in the network (Newman, 2010).

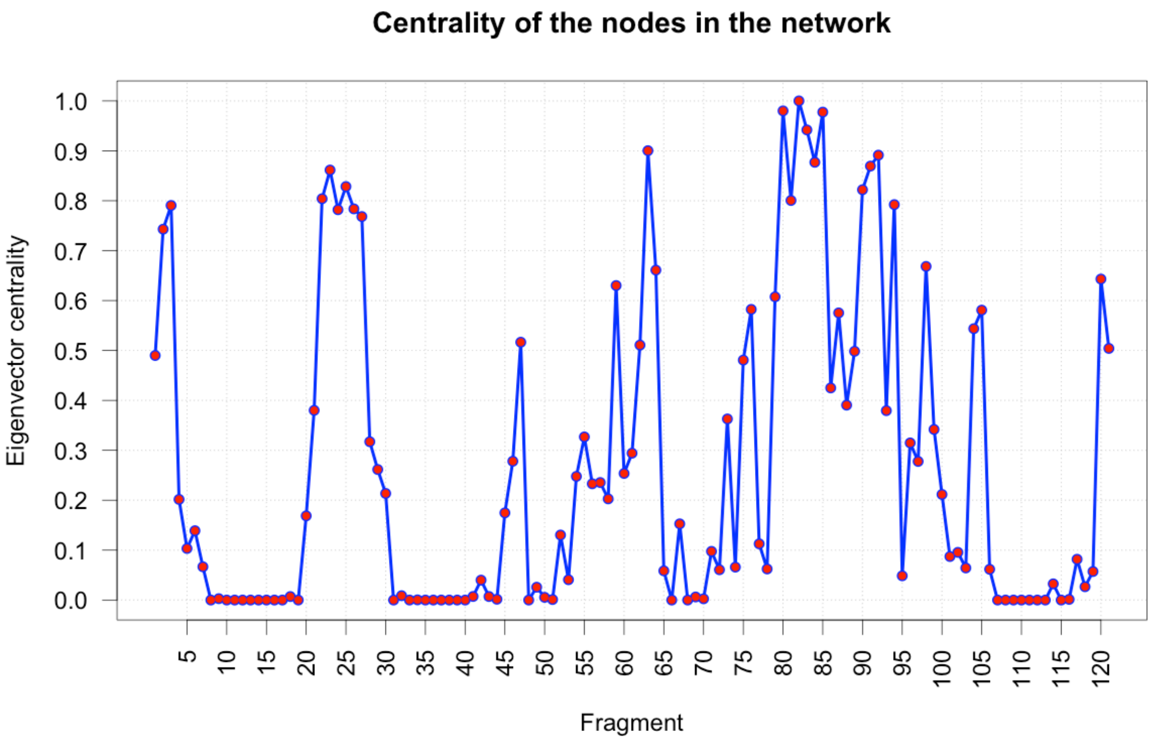

T04.LF_graph_centrality.png (generated with 04_network_analysis.R)

05 functional analysis

This exercise shows how to perform a functional analysis using as a functional index (*f*) the projection of the trajectory onto the first Principal Component obtained from a PCA (Amadei, et al., 1993) of C^{α} positions. For convenience of the user the PC analysis was pre-performed using the GROMACS commands `g_covar` and `g_anaeig` (see GROMACS documentation for details). The correlation between the local conformational changes of a single position (Fragment 80) and *f* is calculated as Mutual Information (MI). To this end the continuous variable *f* is discretized to find the best partition (Pandini, et al., 2012).

```
> cd 05_functional_analysis
> ../../src/g_sa_analyze -sa T00.lf_str.out -value T00.PC1.xvg -n T00.F80.ndx -MIout
  T05.lf_MI.F80-PC1.out -MIxvg T05.lf_MI.F80-PC1.xvg -MIlog T05.lf_MI.F80-PC1.log
```

This generates a few output files, namely:
- T05.lf_MI.F80-PC1.log — summary statistics table with MI and H values (plain text)
- T05.lf_MI.F80-PC1.out — table of *f*, discretized *f* and SA conformations for F80 (plain text)
- T05.lf_MI.F80-PC1.xvg — table of *f*, discretized *f* and SA conformations for F80 (XVG xmgrace format)

A plot can be generated to show the evolution of *f* during the simulation time and how this variable has been discretized. The binning of *f* is reported on the y axis as Partition n.

```
> R CMD BATCH 05_plot_MI_over_time.R
```

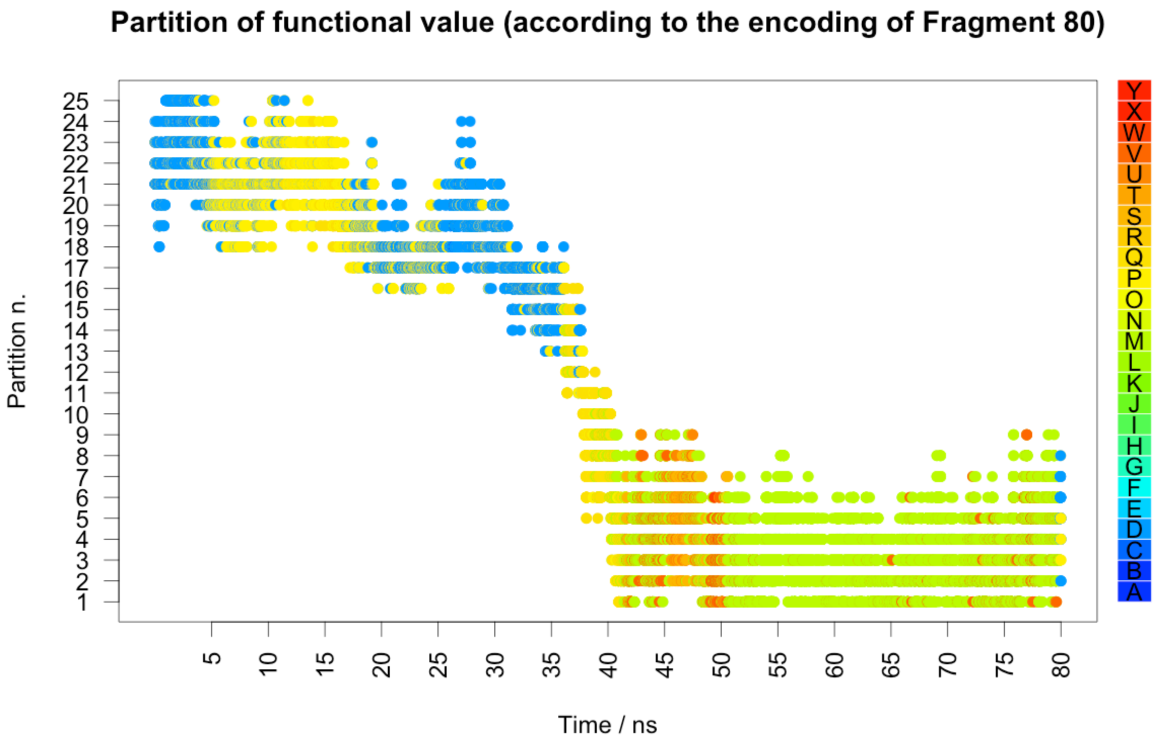

T05.lf_MI.F80-PC1.png (generated with 05_plot_MI_over_time.R)

The functional analysis can be performed for all the fragments in the protein. Then the correlation between *f* and the conformational changes at each fragment position can be plotted to identify putatively interesting regions for further analysis. The correlation is reported as normalized Mutual Information (MI^n) calculated as the ratio between the Mutual Information (MI) and the Joint Entropy (H) as described in (Pandini, et al., 2012).

```
> R CMD BATCH 05_plot_MI_SA-PC1.R
```

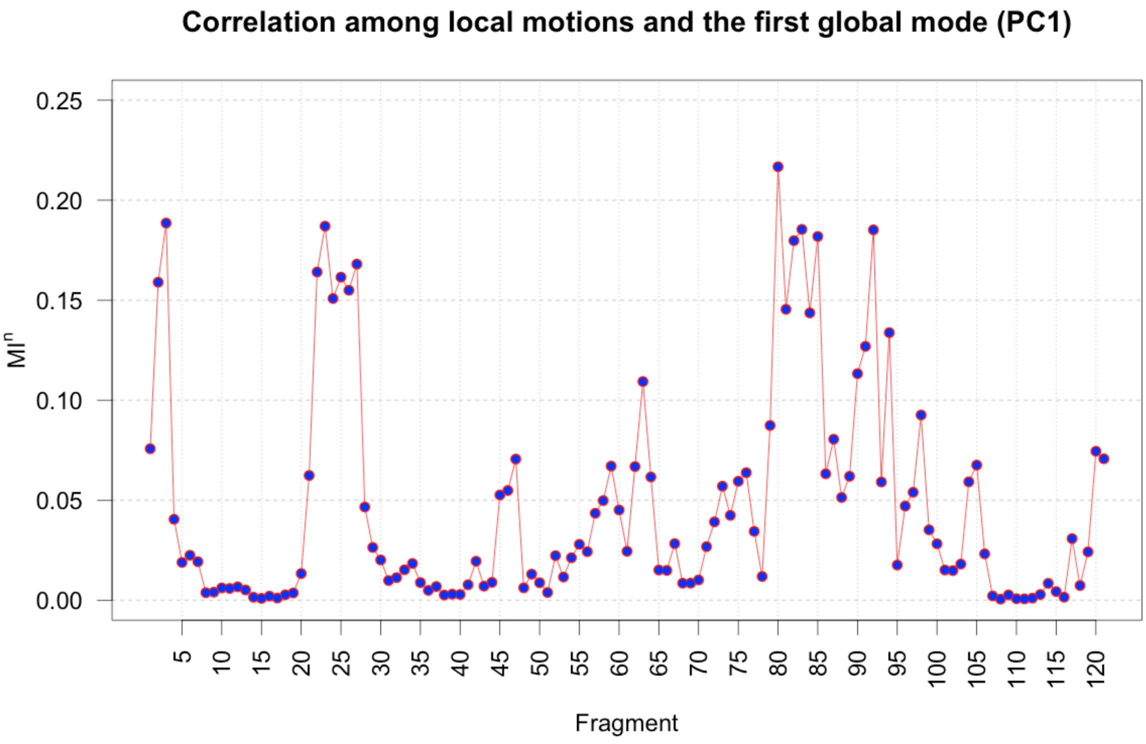

T05.MI_SA-PC1.png (generated with 05_plot_MI_SA-PC1.R)

References

Amadei, A., Linssen, A.B. and Berendsen, H.J. (1993) Essential dynamics of proteins, *Proteins*, **17**, 412-425.

Chennubhotla, C. and Bahar, I. (2007) Signal propagation in proteins and relation to equilibrium fluctuations, *PLoS Comput Biol,* **3,** 1716–1726.

Fanelli, F. and Seeber, M. (2010) Structural insights into retinitis pigmentosa from unfolding simulations of rhodopsin mutants, *FASEB J.,* **24,** 3196–3209.

Genoni, A., *et al.* (2010) Computational study of the resistance shown by the subtype B/HIV-1 protease to currently known inhibitors, *Biochemistry,* **49,** 4283–4295.

Ghosh, A. and Vishveshwara, S. (2007) A study of communication pathways in methionyl- tRNA synthetase by molecular dynamics simulations and structure network analysis, *Proc. Natl. Acad. Sci. U.S.A.,* **104,** 15711–15716.

Grant, B.J., *et al.* (2006) Bio3D: An R package for the comparative analysis of protein structures, *Bioinformatics* **22**, 2695-2696.

Morra, G., Verkhivker, G. and Colombo, G. (2009) Modeling Signal Propagation Mechanisms and Ligand-Based Conformational Dynamics of the Hsp90 Molecular Chaperone Full-Length Dimer. *PLoS Comput Biol* **5,** e1000323.

Newman, M. (2010) *Networks: An Introduction*. Oxford University Press, Oxford, UK.

Pandini, A., Fornili, A. and Kleinjung, J. (2010) Structural alphabets derived from attractors in conformational space, *BMC Bioinformatics*, **11**, 97.

Pandini, A.*, et al.* (2012) Detection of allosteric signal transmission by information-theoretic analysis of protein dynamics, *FASEB J.*, **26**, 868-881.

Pronk, S.*, et al.* (2013) GROMACS 4.5: a high-throughput and highly parallel open source molecular simulation toolkit, *Bioinformatics*, **29**, 845-854.

R-Development-Core-Team (2010) R: A Language and Environment for Statistical Computing. Vienna, Austria.

Sethi, A. et al. (2009) Dynamical networks in tRNA:protein complexes, *Proc. Natl. Acad. Sci. U.S.A.*, **106,** 6620–6625.

Shannon, P.*, et al.* (2003) Cytoscape: a software environment for integrated models of biomolecular interaction networks, *Genome Res*, **13**, 2498-2504.

Van Der Spoel, D.*, et al.* (2005) GROMACS: fast, flexible, and free, *J. Comput. Chem.*, **26**, 1701-1718.
