# Supplementary figures and images for: GSATools: analysis of allosteric communication and functional local motions using a structural alphabet
Source: Bioinformatics. 2013 Jun 5;29(16):2053–5. doi: 10.1093/bioinformatics/btt326 (PMC3722520; doi:10.1093/bioinformatics/btt326)

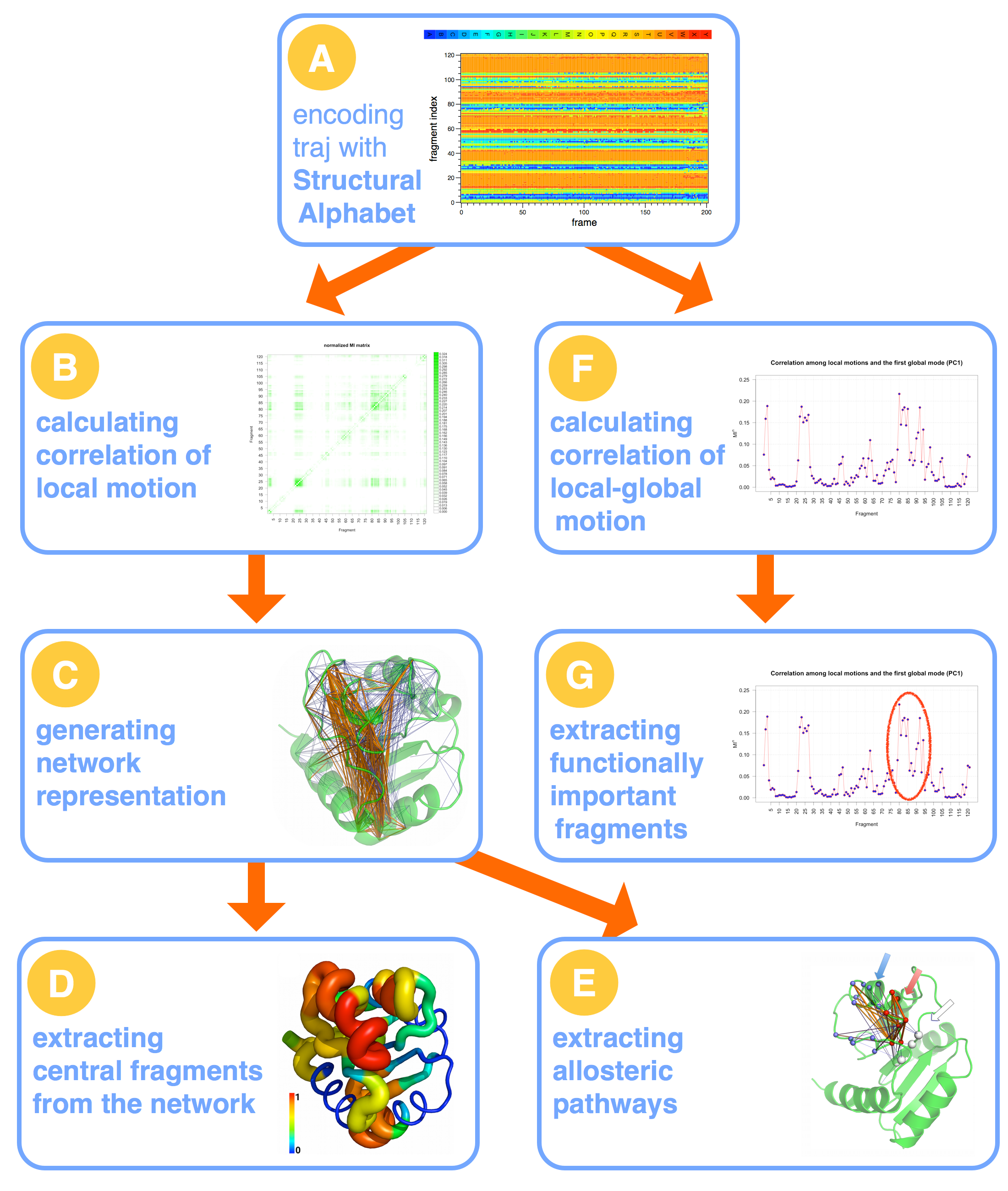

Supplement: Supplementary Data [file supp_btt326_APandini_SupplementaryData_Figure1_HighRes.tif]
